# LOSS NEGATIVELY IMPACTS A HEALTHY LIFE IN US ADULTS: FINDINGS FROM THE HEALTH AND RETIREMENT STUDY

**DOI:** 10.1093/geroni/igz038.3346

**Published:** 2019-11-08

**Authors:** Justin B Ingels, Toni Miles

**Affiliations:** 1 College of Public Health, University of Georgia, Athens, Georgia, United States; 2 University of Georgia College of Public Health, Athens, Georgia, United States

## Abstract

Previous research reports that the loss of a loved one increases the risk of mortality and physical and mental health problems. Using data from the 2004 to 2014 waves of the Health and Retirement Study, we estimate the years of healthy life (YHL) from 2004 to death for each respondent. YHL is based on the combination of years lived between 2004 and 2014, a projection of years beyond 2014, and self-rated health. Regression models stratified by age and gender were developed with the loss of a parent or spouse as the primary exposure and YHL as the dependent variable. Annual estimates of the total YHL lost associated with bereavement were based on these regression analyses and US Census data. Models reveal a strong dose-relationship between YHL lost and the number of losses. In total, the annual YHL lost associated with loss in US adults between 50 and 84 years of age is estimated at 2.0 and 1.6 million for men and women, respectively. Nearly three-fourths of the annual YHL lost are associated with adults younger than 65. Interaction analyses suggest that increasing physical activity has the greatest impact on reducing YHL lost in those with the greatest number of losses, one to two YHL per person. Understanding the full impact of loss on the lives of adults is an important step toward framing loss as a public health issue, especially for middle-aged adults. Results suggest that physical activity should be an important aspect of bereavement interventions.

